# Occupational mental health of non-family members in family firms: Evidence from Pakistan

**DOI:** 10.3389/fpubh.2022.961553

**Published:** 2022-09-26

**Authors:** Khalid Khan, Umm- e-Habiba, Zara Sabeen, Muhammad Waseem

**Affiliations:** ^1^Department of Business Administration, Federal Urdu University of Arts, Sciences and Technology, Islamabad, Pakistan; ^2^Department of Management Sciences, Bahria University, Islamabad, Pakistan; ^3^Department of Business Administration, International Islamic University, Islamabad, Pakistan

**Keywords:** occupational mental health, family—owned business, psychological distance (PD), psychological safety, pro-active personality

## Abstract

Family-owned firms display distinct dynamics as compared to other firms. Consequently, the outcomes and consequences of these dynamics are also expected to be different. The aim of this study was to explore the impact of perceived employee-organization psychological distance (EOPD) on the occupational mental health (OMH) of the employees. Considering the complexities associated with employee–employer relationship, the study also investigated how this relationship between PD and OMH might be mediated by psychological safety (PS) perceived by the employees. Furthermore, the study also included proactive personality (PP) as a potential moderator of the relationship between PD and OMH. Results using SEM and fsQCA show a partial mediation effect on psychological safety. The study contributes by examining the distinct nature of family firms and their impact on the mental health of non-family member employees. This study contributes to the family firm literature by adopting a novel methodological approach to unveil the complexity behind the relationship between employees and owner-employers of family firms.

## Introduction

There has been a sustained increased interest in understanding occupational mental health (OMH) from both the academic and practitioners' perspectives. OMH has become a major concern for organizations that are finding it hard to retain talented employees who are either underperforming due to mental health issues or have decided to leave their jobs based on these concerns (1, 2). The effects of OMH are also self-reinforcing; any drop in productivity due to mental health issues will lead to further mental health deterioration as the pressure starts to build to bridge the productivity deficit (3). The severity of negative work-related outcomes associated with OMH has been exacerbated by the recent COVID-19 pandemic (4). Although OMH has received wide coverage in the broader industrial/organizational psychology literature, the convergence of OMH in the literature focused on the operations of family-owned firms is scant.

Within family firms, specifically those of mid-to-large size, most of the employees are non-family members. These employees have limited access to the organizational management both within the context of business and outside of it, as compared to the immediate or extended members of the family owning the firm. This increases their psychological distance from the management (5). The proximity to a firm's leadership has a greater effect in firms where leadership assigns greater values to the family relationship such as those that exist in collectivistic societies (6). Furthermore, research indicated that family and non-family members of family firms vary in their level of sense of ownership of the firm (7, 8). This can also be accounted for by the close psychological distance of the family firm from the family members than perceived by the non-family members.

This study contributes to the literature on OMH by exploring how it is affected by EOPD. It is proposed that the effect of EOPD on OMH is indirect and that EOPD has a negative effect on psychological safety (PS). A reduction in PS is linked with an increase in OMH. Furthermore, it is proposed that the effect of PD on PS will be mediated by the employee's proactive personality. For the purpose of this proposed study, the operationalization of EOPD proposed by Chen and Li (9) will be employed, who view EOPD as the combination of spatial distance (this can be regarded as the geographic distance – relevant for remote workers), temporal distance (amount of time spent by an individual with an organization), social distance (the distance between a focal person and other organizational foci), expectation (regarding the trajectory of the organizational decisions and its future course of action), and emotional belonging (emotional attachment to the organization). This study also postulates that the proactive personality will moderate the relationship between psychological distance and psychological safety. Specifically, this study will be interested in understanding how individuals with varying levels of pro-activeness and psychological distance develop perceptions of psychological safety. This moderation is grounded in previous research on psychological safety.

## Literature review

### Psychological distance

According to the construal level theory, people create preferences based on their interpretations of events rather than the events themselves (10). Their mental image is influenced not only by the events' actual characteristics but also by their psychological distance from them. According to CLT, if an event occurs far away, it is assumed to be interpreted at a high level in comparison to the proximal event. A central tenet of CLT is that when people experience psychological distance, they alter their interpretations moving from abstract to concrete. These interpretations are usually based on three criteria: if it is distant from oneself rather than close (temporal distance), if it is meaningful to a person dissimilar to oneself rather than similar (social distance), and if it is unlikely rather than likely to occur (hypothetical distance) (10).

Several lines of evidence have established individuals assign a lower probability perceived to causes that are psychologically distant from them (e.g., climate change). It is for this reason that psychological distances are listed as a major cause for the lack of interest among the general public in causes that might impact them at a distance rather than sooner (11). Furthermore, research suggests that increasing psychological proximity to a cause is the greatest technique for encouraging prosocial behaviors (12, 13). Similarly, employees who feel psychologically distant from their managers are less likely to feel negative emotions and risk. The more the psychological proximity, the lesser is the perception of risk and more of safety. In family-owned firms where the employees perceive themselves to be less distant from the managers, the employees feel more comfortable and safe and measure better on wellbeing scales. Hence, they are less likely to endure stress and anxiety associated with weaker relationships.

Psychological proximity is supposed to trigger several mechanisms, including unpleasant emotions (anxiety, rage, grief, or remorse), a more tangible understanding of the implications, and sensitivity and urgency about the situation. It also helps individuals feel more worried and willing to act and engage in prosocial activity in line with their beliefs (14).

However, in case of employee–employer relationship, the employee's psychological proximity and sense of connectedness increase the sense of safety by improving the dyadic relationship. Proximity selection is not always effective (15) and can potentially detract from behavioral intentions (16).

Being psychologically near causes the individual to focus on the feasibility (10) of the prosocial behaviors to be performed, resulting in an overestimation of the costs and an underestimation of the benefits, which are rarely instantaneous. The strategy of increasing psychological distance from the cause may be relevant because it allows the consumer to broaden his or her horizons by focusing on global values (e.g., environmental protection) and to want to act positively in relation to those values (16).

The psychological distance can also decrease desirability for future rewards and weaken positive affective reactions like hope (17), which hinders problem-solving and group action (18). Extending the above to the employees of family firms, they are expected to develop positive outcome expectations and rewards from the managers with whom they perceive to be psychologically more proximal (low psychological distance), and this should result in greater perceived psychological safety.

### Psychological safety and occupational mental health

Employees who view their organization as secure, supportive, and open to receiving new ideas are more likely to reciprocate with an increased level of trust in the organization. Trust is at the core of the psychological safety (PS) construct introduced by Edmondson (19). PS is a measure of the willingness of individuals to take interpersonal risk in a group setting (19). Edmondson (19), while distinguishing between the constructs of general trust and PS, explained that their conceptualization of the PS has more to do with group norms and beliefs than individuals. As such, PS is an evaluation of the group climate to encourage experimentation and voice without the fear of reprisal or any other negative consequence.

Shain et al. (20) identified five factors on which PS can be measured, and these include job demands and requirements of effort, job control or influence, reward, fairness, and support. According to Shain et al. (20), psychological safety is negatively affected when workers are assigned tasks that are beyond their capacity, when they are offered no discretion over the task that they have to perform, when rewards for effort are withheld, when due process is not followed, and when resources such as information required to perform the assigned tasks are withheld from them. More importantly, Shain et al. (20) argued that negative effects of PS can spillover to the broader society outside of the work settings.

In their study, Erkutlu and Chafra (21) contrasted workplaces with high and low PS. The report found that in work environments with low PS, employees manage their voices according to the context of the group rather than expressing their true beliefs. They are less likely to ask for resources to complete their tasks and are more likely to overlook problems rather than report them. The results from this study indicate that all these negative outcomes associated with PS weigh heavy on the employee's mental wellbeing.

Newman et al. (22) conducted an exhaustive review of the PS literature and urged to focus explicitly on characteristics that tap into team members' wellbeing and mental health. They also suggested examining performance-related variables. A key finding from their study was that most of the extant literature PS was from research in the fields of sports and exercise sciences, which place a high importance on the criticality of mental health of individuals (23, 24). Recently, the literature on PS has also permeated the literature on work-related wellbeing in general and studies conducted in this regard are indicating that, as in sports teams, work-based teams also benefit from PS and it improves the OMH of the team members (22). In their conclusion, Newman et al. (22) encouraged future research in PS and deemed it as a significant area of research.

The positive impact of PS on work-related behavior has been reported by numerous studies, such as that by Ahmad et al. (25), who reported that employees working in an environment with high PS are more likely to express their true selves to the group members. Similarly, (26) postulated that psychological safety is a fundamental prerequisite for enhancing employee creativity. Yi et al. (27) reported that psychologically safe work environment is necessary for employees to participate in risky and creative jobs. Moreover, employees' perception of their workplace as safe is a significant motivation for them to be their genuine selves without fear (28). Furthermore, an organization's preference for considering the interests of third-party stakeholders (third-party justice) inflates employees' perceptions that their organization is not self-centered and cares equally for all, which is consistent with third-party justice, and it strengthens their sense of psychological safety (29). Employees are also major stakeholders; thus, they are required to foster the notion that their company is a safe place to work, which will improve their psychological safety perception. In earlier studies, Edmondson (19) discovered that psychological safety is a requirement for organizational learning capability. Other researchers, such as Hur et al. (30) and Ahmad et al. (25), revealed that a safe work environment reduces anxiety, which further stimulates employee creativity. In a recent meta-analysis of 117 studies (including more than 22,000 people) found that psychological safety is linked to a variety of outcomes at both the individual and group level, including communication, work engagement, task performance, and satisfaction (31). The rationale for positive outcomes concerning psychological safety allows members to seek and provide honest criticism from others, collaborate, express their thoughts, and try out new ways to old ones (22). This study will contribute to the literature on PS by specifically focusing on family firms, which provide a unique perspective considering that non-family employees have work with family-based ownership and management of the firm.

### Moderating role of proactive personality

Proactive personality is considered as a personality disposition related to an individual's propensity to take initiative to instigate change in his/her environment, situations, and activities (32, 33). According to Bateman and Crant (32), individuals who exhibit a proactive personality are likely to be “unconstrained by situational forces and who effects environmental changes” (p. 105). In organizational settings, this disposition is considered as a significant indicator of an employee's ability to exert effort to improve their contributions to the workplace (34).

Proactive employees are highly motivated, self-directed, and self-reliant, and they contribute to efforts to bring about changes in the organization (35, 36). Employees with a proactive personality are less vulnerable to social stimuli (35); they initiate more proactive behaviors and rely less on cues originated from other sources in addressing work-related problems (37). Furthermore, proactive employees are more likely to put forward alternative ideas to improve work practices, show robust commitment toward achieving goals, demonstrate high effort and performance (34), and are less reliant on their leaders (38). The primary objective of this study was to determine how perceptions regarding EOPD might affect OMH. The objective of the study with regard to proactive personality types was to determine how this relationship between EOPD and OMH might differ for individuals with high vs. low proactive personality types. The study proposes that employees with high proactive personalities will be more tolerant of psychological distance as compared to individuals who measure low on the proactive personality type scale. The implication of this is that individuals with high proactive personality type will experience less change in their OMH with an increase in their perceived EOPD. This assertion is supported by previous research that identifies low proactive employees as being less likely to take initiatives because they tend to doubt their capacity to influence the workplace and rely more on other sources for information (35) and, thus, will face reduced OMH. Thus, in line with these arguments, we hypothesized that proactive personality moderates the relationship between psychological distance and OMH in such a way that the relationship is weaker when proactive personality is high.

## Methodology

### Questionnaire design and data collection

The data are collected from two Pakistani cities, namely, Islamabad and Rawalpindi. The questionnaire comprised of five parts including demographic variables such as age, gender, education, and department. The second part consisted of psychological distance (PD), psychological safety (PS), proactive personality (PP), and occupational mental health. The survey was conducted from February to March 2022. The questionnaire was self-administered. The sample of the study was based on convenience sampling. Considering the research objective of this study, employees working in family-owned firms were mainly asked to participate in the survey. A total of 3,000 questionnaires were distributed, and 252 were received. Out of which, 214 questionnaires had valid responses and were used for data analysis.

The instrument used for the study was a structured questionnaire consisting of items from multiple sources. For the dependent variable, i.e., OMH, a five-item scale developed by Shamasunder et al. (39), known as the GHQ (General Health Questionnaire), was used. Psychological distance is measured using the six-item scale developed by Chan and Li (9). A seven-item scale developed by Edmondson (19) for psychological safety and a seven-item scale developed by Bateman et al. (32) were used for proactive personality measurement. All these measures consist of the five-point Likert scale.

### Data analysis

In recent literature, PLS-SEM has been used in various disciplines, including accounting (40), human resource management (41), knowledge management (42), corporate social responsibility (43), technological forecasting (44), and management (45). PLS (partial least square) is a composite approach to SEM (structural equation modeling), which allows the analysis of complex models with latent constructs from a prediction perspective (46). PLS-SEM produces mean effects that quantify the average impact of every independent variable (i.e., exogenous variable) on dependent variable [i.e., endogenous variable; (47, 48)]. However, few researchers demonstrated that a mean-centric approach to estimation does not provide complete picture (49–51). To address this gap, researchers have called for using asymmetric approaches that analyze every observation as an individual case instead of treating them as a variable. Following an asymmetric approach, the objective is to explore combinations of independent variables (known as conditions) on the outcome (52).

Ragin (53) presented a prominent approach as a standard tool for asymmetric analysis, known as fuzzy set qualitative comparative analysis (fsQCA) (54). Researchers have observed an exponential increase in fsQCA application in various disciplines (51, 55–62). Many of the mentioned studies have analyzed multi-item constructs. To do that, researchers usually average the items in a set. In contrast, PLS-SEM accounts for measurement error, increasing the validity and reliability of the estimates (47). PLS-SEM also provides some additional information which can be clearly better compared to average scores (63). In doing so, combining PLS-SEM and fsQCA provides assessment facilitation for model predictive power grounded in theory and logic (64).

Many research studies have utilized PLS-SEM and fsQCA in twins. The aim of this study was to jointly apply PLS-SEM and fsQCA (47, 65–67).

### Model assessment using PLS-SEM and fsQCA

#### SEM-PLS analysis

PLS-SEM follows 2-step process. In step 1, PLS-SEM estimates and evaluates the measurement model, which is related to variable measures. Moreover, the structural model establishes validity and reliability, and then the model focuses on its explanatory and predictive power. In step 2, researcher extract latent variables from PLS-SEM analysis, and it helps in explaining the relation between hypotheses, *r*^2^ (combined effect on dependent variable), and *f*^2^ (individual effect of every independent variable on dependent variable). To estimate the path model, SmartPLS3 is used (48). The fsQCA analysis was carried out in Rstudio (68).

The PLS path model shows all item-loadings are above 0.7, supporting the reliability (69). The internal consistency reliability of all constructs falls between 0.7 and 0.95, which is acceptable (70). Moreover, the results indicate that the AVE is >0.5, indicating an acceptable range of convergent validity for all variables. To establish discriminant validity, the HTMT criterion is used. Based on bootstrapping of 5,000 subsamples and a percentile approach, the study confirms that the HTMT value of all constructs is significant at *p* < 0.05, which is lower than the threshold value of 0.85, thus establishing discriminant validity (71).

To assess the structural model, again based on bootstrapping, the path coefficients are checked for significance, the endogenous construct *r*^2^ values and their *f*^2^ effect are determined in Table 1 (70). The R-squared value for occupational mental health was 0.220. Overall, this study found support for all the hypotheses. The study presents that psychological distance is positively related to psychological safety (β = 0.227, *p* = 0.000), thereby supporting hypothesis one. Proactive personality is significantly related to psychological safety (β = 0.280, *p* = 0.000). Psychological safety is positively related to occupational mental health (β = 0.468, *p* = 0.000).

**Table 1:** Independent variable effect size.

**Variables**	**Effect size (f^2^)**
PD	0.122
PS	0.282
PP	1.134

PD, psychological distance; PS, psychological safety; PP, proactive personality.

After testing for hypotheses, we further checked for the mediation role of psychological safety in the proposed model. The mediation results for the study are significant. Psychological safety mediates the relationship between psychological distance and occupation mental health (β = 0.324, *t* = 7.08, *p* = 0.000). The moderating role of proactive personality is significant at 10%.

Furthermore, to assess the model's predictive power for OMH, PLS_predict_ procedure was used with 10 repetitions (72). First, we assessed the PLS path model samples' indicator as evidenced in Q${}_{\text{predict}.}^{2}$ We find that the value for Q${}_{\text{predict}}^{2}$ for all OMH constructs was >0 (Table 2). Then, the root mean squared error (RMSE) was generated by PLS-SEM-based estimates with a linear benchmark model (73). Table 3 represents PLS_predict_ results. The results show that the RMSE value for the OMH construct is lower for PLS-SEM than for the linear model. Altogether, the results indicate that the PLS model has moderate predictive power for OMH.

**Table 2:** Path coefficients and their significance.

**Hypothesis**	**Path coefficient**	***t*-value**	**Significant at 5%**
PD → PS	0.227	4.636	0.000
PP → PS	0.280	14.551	0.000
PS → OMH	0.468	5.222	0.000
PD → PS → OMH	0.106	7.082	0.000
PP* PD ->OMH	0.046	1.411	0.080*

PD, psychological distance; PS, psychological safety; PP, proactive personality; OMH, occupational mental health; ^*^, 10% significance level.

**Table 3:** Results of predictive power assessment using PLS_predict_.

	**RMSE**	
**Hypothesis**	**PLS-SEM**	**Linear Model**
OMH1	1.203	1.253
OMH2	0.909	0.954
OMH3	1.265	1.358
OMH4	0.798	0.813
OMH5	0.906	0.905
OMH6	0.914	0.918
OMH7	0.867	0.861
OMH8	0.831	0.823
OMH9	0.882	0.879

OMH, occupational mental health.

#### Fuzzy set qualitative comparative analysis

The data are further used to perform fsQCA. The data are then calibrated for the conditions (IV) and the outcome (DV). Calibration was performed using the Total Fuzzy and Relative (TFR) method. TFR uses rank order and is used to calibrate Likert scale data. To run TFR in the R-studio, the “Calibrate” command is used to calibrate data. After calibration, the data are further tested for NCA. In R-studio, we used the command “pof” to check inclusion and “RON” of every condition on outcome. Researchers use different threshold criteria for social sciences, i.e., 0.8, 0.85, 0.90, and 0.95 (4, 52, 61, 74, 75). For any condition to be necessary, the inclusion score should be >0.8 (50). Proactive personality (PP) appeared to be a necessary condition for the presence of OMH. However, there is no necessary condition for the absence of OMH (~OMH) as presented in Table 4.

**Table 4:** Necessity table.

**Conditions**	**OMH**	**~OMH**
PD	0.636	0.720
~PD	0.614	0.614
PS	0.764	0.729
~PS	0.473	0.445
PP	0.847	0.731
~PP	0.285	0.588

PD, psychological distance; PS, psychological safety; PP, proactive personality; ~, absence of condition.

The next step involves the analysis of the truth table. The truth table is used for logical minimization that helps the researcher to generate a solution model. For generating a truth table in R-studio, the Truthtable command computes all the possible configurations Every row represents all possible combinations for the outcome. “0” indicates the absence of a condition, and “1” indicates the presence of a condition. The column “out” explains the presence and absence of output in the form of “0” and “1.”

After generating the truth table, the data are further analyzed to generate the solution model. R-studio utilizes “Quine-McCluskey” algorithm to generate three solutions, named as, complex, parsimonious, and intermediate. The intermediate and parsimonious solution is always part of a complex solution. This solution model consists of “peripheral conditions” and “core conditions” (52). Core conditions are present in intermediate as well as in parsimonious solution, but “peripheral conditions” are only present in intermediate solutions (52, 61). Generally, the solution model is presented in Fiss chart (Table 5) representing black circles (•) and crossed circles (⊗). Black circles indicate presence while crossed circles represent the absence of the condition. Moreover, the large circles represent core condition, while small circles indicate peripheral condition, and blank space refer to “do not care” condition.

**Table 5:** Fiss chart for high OMH.

	**Causal**		**Metrics**				
	**conditions**	**PS**	**Raw**	**Unique**	**Consistency**	**Overall**	**Overall**
**Solution**	**PD**	**PP**	**coverage**	**coverage**	**Consistency**	**solution coverage**	**solution consistency**
1	⊗	•	0.762	0.208	0.868	0.844	0.714
2	⊗	•	0.623	0.403	0.864		

•, the presence of condition; ⊗, the absence of condition; blank space, do not care.

The fsQCA analysis returned two paths leading to high OMH. Solution 1 that illustrates ~PD^*^PS will lead to high OMH. Solution 2 that represents ~PS^*^PP will lead to high OMH.

## Discussion

This study aimed at finding out the impact of psychological distance from the family member owner on the mental health of non-family member employees in the family firms. Moreover, the target included checking if psychological safety mediates the relationship between psychological distance and mental health. Employee's proactive personality is taken as a moderator that, along with the perception of psychological distance, was proposed to enhance the feeling of psychological safety, hence leading to better mental health of the employees.

The results of the study confirm the partial mediation of psychological safety between psychological distance and mental health. Employees who perceive psychological distance from the firm's family member owner feel psychologically safer and thus have better mental health. The moderating role of proactive personality, however, was not validated in our research. The reason for perceptions of safety when there is more perceived psychological distance is as per the CLT. Employees at a psychological distance have an abstract image of reality due to being at a high construal level and hence are less sensitive to the risks and threats attached to it (15). This inability to sense the danger or possibility of a negative outcome of any action makes him/her feel psychologically safer.

In this study, the role of personality is found to be less important in strengthening the relationship between psychological distance and psychological safety. Proactive personality very slightly affects the probability of feeling safer while the non-family member employee is at a greater psychological distance from the family member owner/manager. That is, the direct relationship between psychological distance and psychological safety is stronger and ultimately leads to better occupational mental health. The study helps us validate the findings using the conventional SEM method along with fsQCA.

The study was conducted as a cross-sectional one. Future studies may utilize time lag data to find out overtime changes in the perceptions and occupational health impacts as the experience of an employee increases with the firm. Further studies may consider different personality traits separately, such as traits mentioned in Big Five model. Similarly, personality may be tested as a mediating variable between psychological distance and psychological safety. Furthermore, occupational consciousness may be considered as an independent variable in the given model.

## Data availability statement

The original contributions presented in the study are included in the article/supplementary files, further inquiries can be directed to the corresponding author.

## Author contributions

All authors listed have made a substantial, direct, and intellectual contribution to the work and approved it for publication.

## Conflict of interest

The authors declare that the research was conducted in the absence of any commercial or financial relationships that could be construed as a potential conflict of interest.

## Publisher's note

All claims expressed in this article are solely those of the authors and do not necessarily represent those of their affiliated organizations, or those of the publisher, the editors and the reviewers. Any product that may be evaluated in this article, or claim that may be made by its manufacturer, is not guaranteed or endorsed by the publisher.
